# Exercise-Dependent effects of substance P deficiency on joint degeneration and inflammation in a surgical mouse model of osteoarthritis

**DOI:** 10.1186/s13075-025-03693-7

**Published:** 2025-12-04

**Authors:** Patrick Pann, Paul Kalke, Verena Maier, Nicole Schäfer, Hauke Clausen-Schaumann, Arndt F. Schilling, Susanne Grässel

**Affiliations:** 1 Department of Orthopaedic Surgery, Experimental Orthopaedics, Center for Medical Biotechnology, University of Regensburg, ZMB im Biopark 1 Am Biopark 9, Regensburg, 93053 Germany; 2 Department of Trauma Surgery, Orthopedics and Plastic Surgery, University Medicine Göttingen, Göttingen, Germany; 3 Center for Applied Tissue Engineering and Regenerative Medicine (CANTER), University of Applied Sciences Munich, Munich, Germany

**Keywords:** Osteoarthritis, Destabilization of the medial meniscus, Substance P, Tachykinin 1, Exercise, Bone, Cartilage, Inflammation

## Abstract

**Background:**

Osteoarthritis (OA) is a chronic degenerative joint disease driven by multifactorial causes, including aging, mechanical stress, and inflammation. Mechanical loading through exercise can either exacerbate or alleviate OA symptoms depending on intensity. Substance P (SP), a neuropeptide involved in inflammation and mechanotransduction, has been implicated in cartilage and bone remodeling. This study aimed to investigate how SP deficiency plus exercise intensity interact to influence disease progression in a surgical murine OA model.

**Methods:**

OA was induced in male wild-type (WT) and SP knockout (Tac1-/-) mice via destabilization of the medial meniscus (DMM). Mice were then exposed to moderate or intense treadmill exercise for up to eight weeks. Cartilage degeneration was assessed histologically using OARSI scoring. Cartilage stiffness was evaluated via atomic force microscopy (AFM), and subchondral and metaphyseal bone morphology was analyzed by high-resolution nanoCT. Serum cytokine levels were measured with multiplex ELISA.

**Results:**

DMM surgery induced OA-like cartilage damage in most groups, and moderate exercise failed to prevent degeneration. However, SP-deficient mice subjected to intense exercise showed preserved cartilage matrix stiffness and morphology comparable to Sham controls. In contrast, SP deficiency as well as intense exercise promoted meniscal ossification and subchondral bone sclerosis, with increased bone volume fraction and trabecular thickness. These changes were consistent with prior findings in SP-deficient mice without exercise. Serum analysis revealed elevated levels of proinflammatory cytokines (e.g., CXCL10, VEGF-A, CCL2, CCL4) in SP-deficient mice after Sham surgery, although these did not correspond to the cartilage degradation timeline.

**Conclusions:**

SP plays a dual role in OA pathogenesis: its absence may protect cartilage from mechanical stress–induced stiffening but also promotes ectopic meniscal ossification and subchondral bone alterations. Additionally, SP appears to modulate systemic inflammatory responses independently of joint degeneration. These findings position SP as a key regulator of neuroimmune and mechanobiological processes in OA and highlight its potential as a therapeutic target for load-induced joint pathology.

**Supplementary Information:**

The online version contains supplementary material available at 10.1186/s13075-025-03693-7.

## Introduction

Osteoarthritis (OA) is a prevalent, long-term degenerative joint disorder that primarily affects adults over the age of 40 [1]. Characterized by progressive cartilage degradation, remodeling of subchondral bone, synovial inflammation, and osteophyte formation, OA leads to pain, joint stiffness, and impaired mobility [2, 3]. Its pathogenesis is multifactorial, with contributing factors including high age, genetic susceptibility, obesity, trauma, and mechanical stress [4]. As a major source of disability worldwide, OA imposes a substantial socioeconomic burden due to increased healthcare costs, reduced productivity, and diminished quality of life [5]. Despite advances in research, no curative treatment exists, and current therapeutic strategies largely aim to manage symptoms rather than alter the course of the disease [2].

Mechanical loading plays a complex role in OA. On one hand, excessive or repetitive mechanical stress—particularly after joint injury—can disrupt tissue homeostasis and contribute to the development of post-traumatic OA (PTOA) [6]. On the other hand, appropriately dosed physical activity is among the most effective non-pharmacological approaches for managing early OA symptoms. Aerobic, resistance, and neuromuscular exercise regimens have consistently demonstrated benefits in terms of pain relief, muscle strengthening, and functional improvement. Systematic reviews confirm the efficacy of both land-based and aquatic exercise programs in reducing OA-related symptoms without worsening joint degeneration [4]. Nevertheless, the biological mechanisms underlying the effects of mechanical stress on OA pathophysiology remain incompletely understood.

Recent findings have underscored the critical role of the sensory nervous system in regulating joint homeostasis and OA progression. Neuropeptides such as substance P (SP) and alpha-calcitonin gene-related peptide (αCGRP), released from afferent nerve fibers innervating the meniscus, synovium, and subchondral bone, act as signaling molecules that influence inflammation, nociception, and tissue remodeling [7]. SP, an 11-amino-acid tachykinin, binds to its high-affinity neurokinin-1 receptor (NK1R), which is expressed not only by neurons but also by joint-resident and bone-related cells, including chondrocytes, osteoblasts, osteocytes, osteoclast precursors, synovial fibroblasts, and bone-marrow–derived macrophages. Through these receptors, SP modulates mechanical responsiveness, cellular proliferation, differentiation, cytokine release, and matrix metabolism, thereby contributing to both bone and cartilage turnover [7, 8]. In chondrocytes, SP–NK1R signaling participates in mechanotransduction: mechanical loading increases NK1R expression and SP release, promoting characteristic membrane hyperpolarization and mechanically induced gene expression [8, 9]. Disruption of this pathway, for example by NK1R inhibition or SP deficiency, impairs chondrocyte responsiveness to mechanical stimulation and may compromise cartilage homeostasis under load [9]. Moreover, in vitro studies have shown that mechanical stimulation induces expression of the tachykinin (TAC1) gene -encoding SP- in OA chondrocytes, whereas this response is absent in chondrocytes from non-OA cartilage [10]. In vivo, SP deficiency has been associated with increased subchondral bone sclerosis and altered joint remodeling in OA models [3]. Together, these findings indicate that SP-NK1R signaling integrates neuronal, inflammatory, and mechanical cues within the joint and may play a dual role in maintaining tissue integrity and driving OA pathogenesis.

Given that physical activity affects both joint biomechanics and neuropeptide signaling, SP may serve as a critical mediator of exercise-induced effects on OA progression. We hypothesize that SP plays an active role in modulating the relationship between mechanical loading and OA pathogenesis. To test this, the present study investigates the impact of SP deficiency in a surgically induced mouse model of OA, which were subjected to forced treadmill exercise. By examining the effects of combined mechanical stress and neuropeptide signaling disruption, this work aims to advance our understanding of SP-dependent mechanisms in joint degeneration and uncover novel targets for therapeutic intervention.

## Materials and methods

### Animals

The origins and housing conditions of the experimental animals were identical to those in our previous study by Muschter et al. 2020 [3]. Male wild-type (WT) C57Bl/6J mice were purchased from Charles River Laboratories (Sulzfeld, Germany) at an age of 8–10 weeks and maintained under standard housing conditions, including a 12-hour light/dark cycle, until the animals reached an age of 12 weeks. Additionally, global Tachykinin 1 knockout mice (Tac1−/−, SP-deficient), matched in age and sex, were generously provided by A. Zimmer from the University of Bonn [11] and subsequently backcrossed onto the C57Bl/6J genetic background. The WT C57Bl/6J mice served as control animals for comparative evaluations. Food and water were available to all mice ad libitum. In total 102 WT and 105 KO animals were used in this study. All experimental protocols were reviewed and approved by the local ethics authority (Regierung von Unterfranken, Bavaria; reference number: RUF-55.2.2–2532.2-2–1253; date of approval: November 5, 2020).

### Destabilization of the medial meniscus

OA was induced through surgical destabilization of the medial meniscus (DMM), following the protocol initially described by Glasson [12]. Considering the hormonal sensitivity of the DMM model, only male mice were employed in these experiments [13]. In brief, mice underwent intraperitoneal anesthesia comprising fentanyl (0.05 mg/kg), medetomidine (0.5 mg/kg), and midazolam (5 mg/kg). A 3 mm longitudinal skin incision was made on the right leg, positioned between the distal patella and the proximal tibial plateau, to expose the knee joint. The joint capsule was subsequently accessed via a 1–2 mm incision just medial to the patellar tendon. Using micro-scissors, the medial meniscotibial ligament was carefully transected to initiate OA. For the Sham-operated control group, the ligament was merely visualized without being dissected. Following the procedure, the incision was closed by suturing, and anesthesia reversal was achieved using Atipamezol (2.5 mg/kg) and Flumazenil (0.5 mg/kg). Postoperative analgesia was administered with buprenorphine (0.1 mg/kg) immediately following surgery, with subsequent doses at 8, 16, and 24 h post-surgery. Animals were permitted unrestricted movement during the recovery period.

### Treadmill exercise

**Fig. 1:**
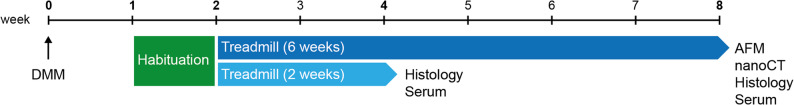
Experimental timeline

Mice underwent treadmill exercise beginning one week post-surgery, initially undergoing a habituation phase for one week on a motorized treadmill (Ugo Basile, Gemonio, Italy). Subsequently, two weeks after surgery, the mice were randomly allocated to either moderate exercise (10 m/min, 5° incline) or intense exercise (16 m/min, 15° incline). Exercise sessions were conducted for 30 min daily, five times per week, for durations of either two or six weeks. Exercise settings were guided by previously published protocols demonstrating either a mitigating (moderate) or aggravating (intense) effect on OA progression [14, 15]. The moderate regime was designed to promote brisk walking without triggering running or jumping, thereby ensuring consistent engagement and minimizing strain. In contrast, the intense protocol pushed animals to the upper limit of sustainable speed over the session duration, with the added incline intended to amplify mechanical loading on the hind limbs. Animals were euthanized with CO_2_ at four and eight weeks post-surgery. Based on previous studies, eight weeks post-surgery were expected to yield measurable yet not excessive cartilage degeneration, allowing differentiation between WT and KO groups. Four weeks post-surgery were included as an intermediate assessment [3, 15, 16]. Blood samples were collected immediately after euthanasia via intracardiac puncture. Knee joint samples were promptly harvested for histological analysis. For atomic force microscopy (AFM) and nano-computed tomography (nano-CT), samples were collected eight weeks after surgery and were prepared as detailed in their respective method descriptions. The experimental timeline is shown in Fig. 1.

### Serum analysis

Eight weeks post-surgery, blood samples were obtained via intracardiac puncture immediately following euthanasia. Serum was isolated by centrifugation, and multiplex immunoassays were conducted to quantify pro-inflammatory and OA-associated factors. Serum analysis utilized a Bio-Plex 200 Multiplex Reader (Bio-Rad Laboratories GmbH, Feldkirchen, Germany) in combination with a Mouse ProcartaPlex Mix&Match 14-plex assay (PPX-14-MX324DH, Thermo Fisher Scientific, Vienna, Austria), measuring the cytokines and chemokines Eotaxin (CCL11), GRO alpha (CXCL1), IFN-γ, IL-1β, IL-6, IP-10 (CXCL10), Leptin, MCP-1 (CCL2), MIG (CXCL9), MIP-1α (CCL3), MIP-1β (CCL4), RANKL, RANTES (CCL5), and VEGF-A.

### Histology and OA scoring

Histological sample preparation and OA scoring were performed as described in our previous study [3]. Following dissection, knee joints were fixed for 24 h in 4% paraformaldehyde/PBS and decalcified for eight weeks in 20% EDTA (pH 7.4). After embedding in paraffin, 6 μm frontal sections were cut using a microtome. To assess cartilage deterioration, six pairs of consecutive sections, each pair spaced 60–90 μm apart (12 sections total) were deparaffinized, rehydrated, and stained using Safranin O, Weigert’s iron hematoxylin, and Fast Green. Two independent investigators, blinded to experimental conditions, evaluated the sections according to OARSI guidelines [17]. In cases where OARSI scores differed by more than 1.5 standard deviations between the two evaluators, a third evaluator performed an additional assessment. Digital images were captured using a BZ-X810 microscope (KEYENCE Deutschland GmbH, Neu-Isenburg, Germany) at 10× magnification. Mean maximum OARSI scores from lateral and medial femoral condyles and tibial plateaus were calculated.

### Indentation-type atomic force microscopy

Atomic force microscopy (AFM) analysis was adapted from the protocol described in our previous study [3], with the use of a newer-generation AFM and microscopy system enabling larger-area mapping and higher vertical tip velocities. AFM was used in quantitative imaging mode (QI™) and was performed on non-fixed native knee tissue samples collected eight weeks post-DMM or sham surgery. The samples were rapidly frozen and cut frontally into 20 μm sections using a cryotome (Leica CM 1950). The sections were collected using transparent adhesive tape (tesafilm Nr.: 57330-00000) to preserve structural integrity and mounted onto glass slides with double-sided adhesive tape (tesafilm Nr.: 56661-00002). QITM measurements employed a NanoWizard IV system (Bruker Nano GmbH, Berlin, Germany) integrated with an inverted optical microscope (Stellaris 8, Leica Microsystems GmbH, Wetzlar, Germany) for precise lateral AFM-tip positioning. The setup was placed on an active vibration isolation table (Accurion, Park Systems Corporation, Korea) within a soundproof enclosure to minimize external disturbances. Measurements used silicone-nitride cantilevers (MLCT, Cantilever F, Bruker) with a spring constant of approximately 0.6 N/m, a tip radius of 20 nm, and a pyramidal shape. Spring constants were individually verified by the thermal noise method [18]. During measurements, tissue sections were submerged in PBS (Bio&Sell (D)PBS, without Ca2 + and Mg2+, Feucht, Germany). Cartilage extracellular matrix (ECM) from superficial, middle, and deep zones of the medial tibia plateau was analyzed immediately after thawing. Force maps (128 × 128 force-indentation curves per 15 × 15 μm² area) were recorded at a vertical tip velocity of 300 μm/s, with three force maps per zone, surgery type, genotype, and exercise condition from three distinct sections per animal. Analysis included extracting Young’s Modulus using the Hertz model (modified for pyramidal indenters, up to 1 μm indentation depth) via JPK Data Processing Software. Error-prone artifacts or cell areas (R² < 0.975) were removed using CANTER Processing Toolbox (https://github.com/CANTERhm/CANTER_Processing_Tool). Finally, stiffness distributions were created, and the two maxima of the bimodal distributions were calculated by fitting a linear combination of two Gaussian distributions to the data [19]. Loparic et al. found a comparable bimodal nano-stiffness in mature articular cartilage and demonstrated that the first peak can be attributed to the proteoglycan phase while the second peak belongs to the collagen fibrils [20].

### NanoCT analysis

NanoCT analysis was adapted from the protocol described in our previous study [3]. Immediately following dissection, knee joint specimens were fixed in 4% paraformaldehyde dissolved in PBS for 24 h and subsequently stored at 4 °C in 70% ethanol. Nano-CT was carried out based on methods previously outlined by Muschter et al. [3]. Briefly, imaging of the knee joints was performed with a Scanco µCT 50 scanner (Scanco Medical, Brüttisellen, Switzerland) under ambient air conditions. The scanning parameters utilized were 90 kVp source voltage and 88 µA current, with beam hardening minimized by employing an aluminum filter of 0.50 mm thickness. All scans were acquired at an isotropic voxel resolution of 6.8 μm and integration time set at 1000 ms, intended to produce detailed three-dimensional visualizations of the femorotibial joints, particularly for the identification of alterations in metaphyseal regions, meniscal ossifications, and osteophyte formation. Reconstruction of nano-CT images was done using Scanco’s proprietary OpenVMS software. Bone morphometric parameters were quantified within two specifically defined regions of interest (VOIs). The initial VOI, encompassing 1.2 mm³, targeted the metaphyseal area 300 μm distal from the epiphyseal line, allowing assessment of sub-articular trabecular morphometry. The second VOI, approximately 0.2 mm³ in size, was positioned within the medial epiphysis between the epiphyseal line and the lower boundary of the subchondral bone plate. For both VOIs, optimized threshold settings compatible with Scanco’s OpenVMS software were selected (lower threshold: 685.3 mg HA/cm³; upper threshold: 3000 mg HA/cm³; Gauss Sigma: 0.8; Gauss Support: 1). Manual contouring excluded the endocortical surfaces in accordance with standard imaging practices. The thickness of the subchondral bone plate was quantified utilizing ImageJ software across three evenly spaced coronal cross-sections of each joint sample, with the presented data representing mean ± SEM values derived from measurements at 60 individual points per condyle across these three planes. Tibial plateau geometry was evaluated by measuring lateral and medial condyle diameters, defined as distances extending from the posterior intercondylar area to their respective lateral or medial prominences. Assessment of heterotopic ossification within the meniscus involved manually delineating anterior meniscal ossicles. VOIs for sham-operated and DMM-operated mice differed due to variations in ossicle formation, measuring approximately 0.4 mm³ and 1.0 mm³, respectively. Quantitative analysis encompassed parameters such as bone volume (BV), bone mineral density (BMD), bone surface (BS), and the bone surface-to-volume ratio (BS/BV) within the anterior meniscus.

### Statistical analysis

Biometric planning was conducted using G*Power 3.1.9 (Heinrich Heine University, Düsseldorf), drawing on data from a prior study by our group involving WT and SP-deficient mice [3]. Sample size calculations for comparisons across multiple groups were carried out using an F-test (one-way ANOVA with fixed effects, omnibus), assuming a statistical power of 80% and a type I error rate of 0.05. To account for multiple comparisons, a Bonferroni correction was applied.

Data analysis and graphical representation were performed using GraphPad Prism version 8.0.2 (San Diego, CA, USA). Outlier detection was conducted with the ROUT method, setting Q = 1% for OARSI scores and Q = 5% for serum data [21]. Differences in OARSI scores between groups based on genotype, surgical intervention, and exercise regimen were assessed using Kruskal-Wallis tests followed by Dunn’s multiple comparisons test. Serum, nano-CT, and AFM data were analyzed by two-way ANOVA with Tukey’s multiple comparison post hoc tests to identify genotype-, surgery-, and exercise-related differences. OARSI scores and serum concentrations are represented as box plots indicating medians with interquartile ranges and whiskers indicating minimum and maximum values. Nano-CT and AFM results are presented as bar graphs showing means ± standard deviations. Representative AFM data distributions are further illustrated using histograms.

## Results

### Tac1 deficiency and exercise intensity modulate OA progression

**Fig. 2:**
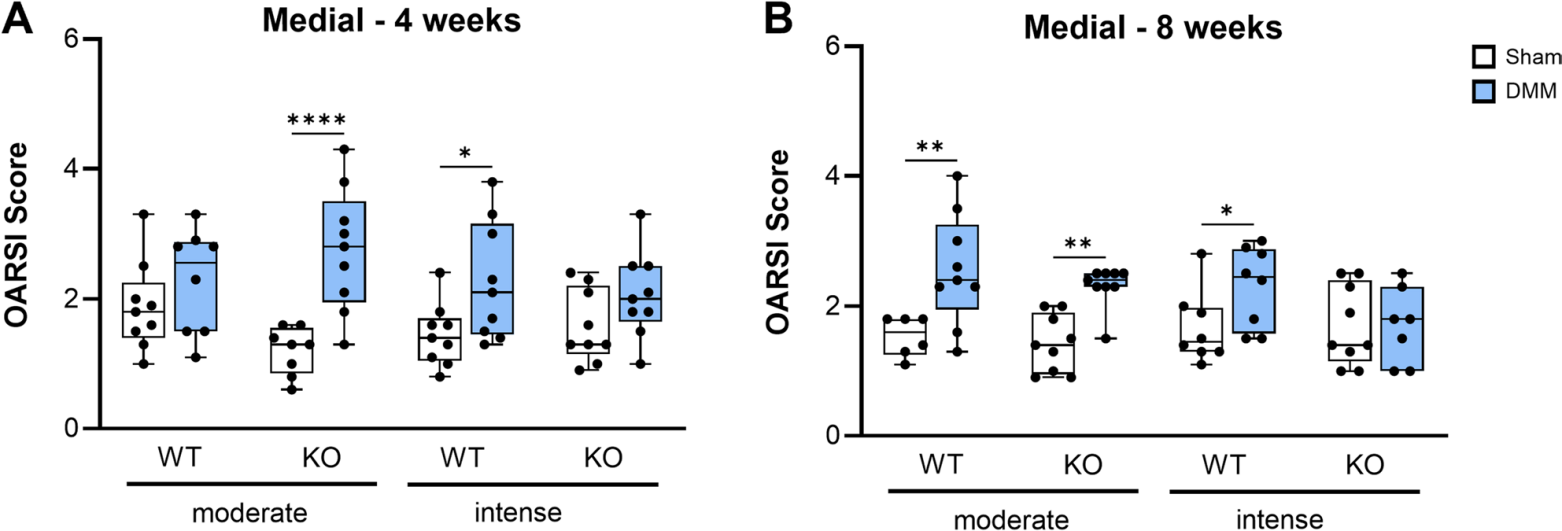
Impact of Tac1 deficiency and exercise intensity on medial cartilage degradation after OA induction. Cartilage was evaluated for grades of destruction according to the OARSI guidelines for murine OA. Cartilage of the right knee joints of WT and KO mice exposed to moderate or intense exercise were graded 4 weeks (**A**) and 8 weeks (**B**) after Sham or DMM surgery. Means of the sum maximal OARSI scores of the medial tibial and femoral cartilage together were compared. Statistical analysis is using Kruskal-Wallis and Dunn’s test for multiple comparisons. * *p* < 0.05, ** *p* < 0.01. *N* = 6–10

OA development, assessed by maximum OARSI scores in the medial compartment, was induced by DMM and influenced by both exercise intensity and Tac1 deficiency at 4 and 8 weeks post-surgery (Fig. 2, Suppl. Figure 1). OARSI scores in Sham-operated mice remained consistently low (1–2) across all groups. At 4 weeks (Fig. 2A, Suppl. Figure 1), DMM KO mice undergoing moderate exercise and DMM WT mice subjected to intense exercise exhibited significantly elevated OARSI scores, which persisted through week 8. In contrast, WT mice under moderate exercise showed delayed OA onset, with significant cartilage degradation not apparent before 8 weeks (Fig. 2B, Suppl. Figure 1). Notably, KO mice exposed to intense exercise maintained OARSI scores comparable to Sham controls with no significant increase, indicating cartilage resistance to OA progression. In the lateral compartment, cartilage integrity was largely preserved regardless of genotype, exercise intensity, or surgical intervention (Suppl. Figures 1, 2). An exception was observed in WT mice under moderate exercise, which displayed lateral cartilage degradation at 8 weeks, comparable to medial compartment involvement.

### Tac1 deficiency prevents cartilage stiffening after OA induction and intense exercise

**Fig. 3:**
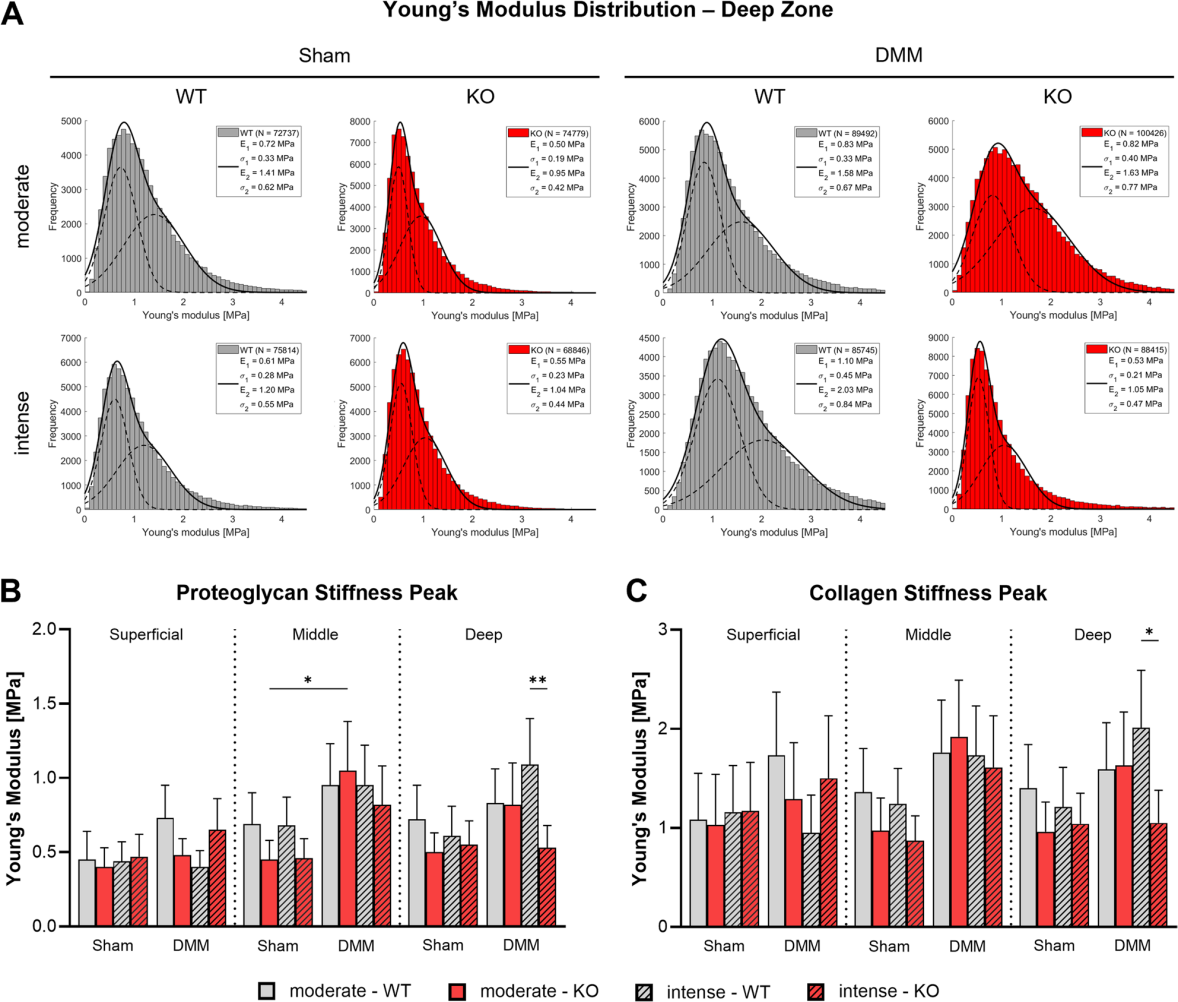
Atomic force microscopy-based analysis of cartilage matrix stiffness in Tac1 deficient mice after OA-induction and forced exercise. Analysis of articular cartilage surface properties of the right knee joint of WT and KO mice exposed to moderate and intense exercise at 8 weeks after DMM or Sham surgery. **A** Histograms of Young’s modulus (stiffness) distribution of the deep zone cartilage matrix (histograms of superficial and middle zone are in the supplementary material). The black line in each histogram represents a fit to the data using a linear combination of two Gaussian distributions. The dashed black lines show the individual Gaussian distributions representing the proteoglycan (E1, σ1) and the collagen (E2, σ2) Young's moduli, respectively, as described in detail in the methods section. *N *= 3. **B**-**C** Mean Young's modulus (stiffness) of the proteoglycan (**B**) and collagen (**C**) peaks of superficial zone, middle zone, and deep zone cartilage. Bars show mean ± standard deviation. Statistical analysis using Two-Way ANOVA and Tukey’s multiple comparisons test. *p<0.05, ** *p*<0.01. *N *= 3

Stiffness of the superficial, middle, and deep zones of the articular cartilage matrix was assessed 8 weeks post-surgery via atomic force microscopy (AFM), analyzing both proteoglycan- and collagen-associated Young’s modulus values (Fig. 3; Suppl. Figures 3, 4). Overall, DMM surgery tended to increase cartilage matrix stiffness, particularly in the middle and deep zones. In the middle and deep zone, KO mice exposed to moderate exercise showed a significant increase in proteoglycan stiffness and by trend also in collagen stiffness, driven by a DMM-induced elevation relative to Sham controls, which in turn exhibited lower stiffness compared to WT Sham mice. In contrast, Tac1 deficiency conferred cartilage matrix protection in the deep and middle zone under intense loading: KO mice subjected to DMM and intense exercise maintained proteoglycan and collagen mediated matrix stiffness values comparable to Sham controls, indicating attenuation of OA-induced matrix stiffening in this compartment.

### Tac1 deficiency enhances OA induced ectopic meniscal bone formation

**Fig. 4:**
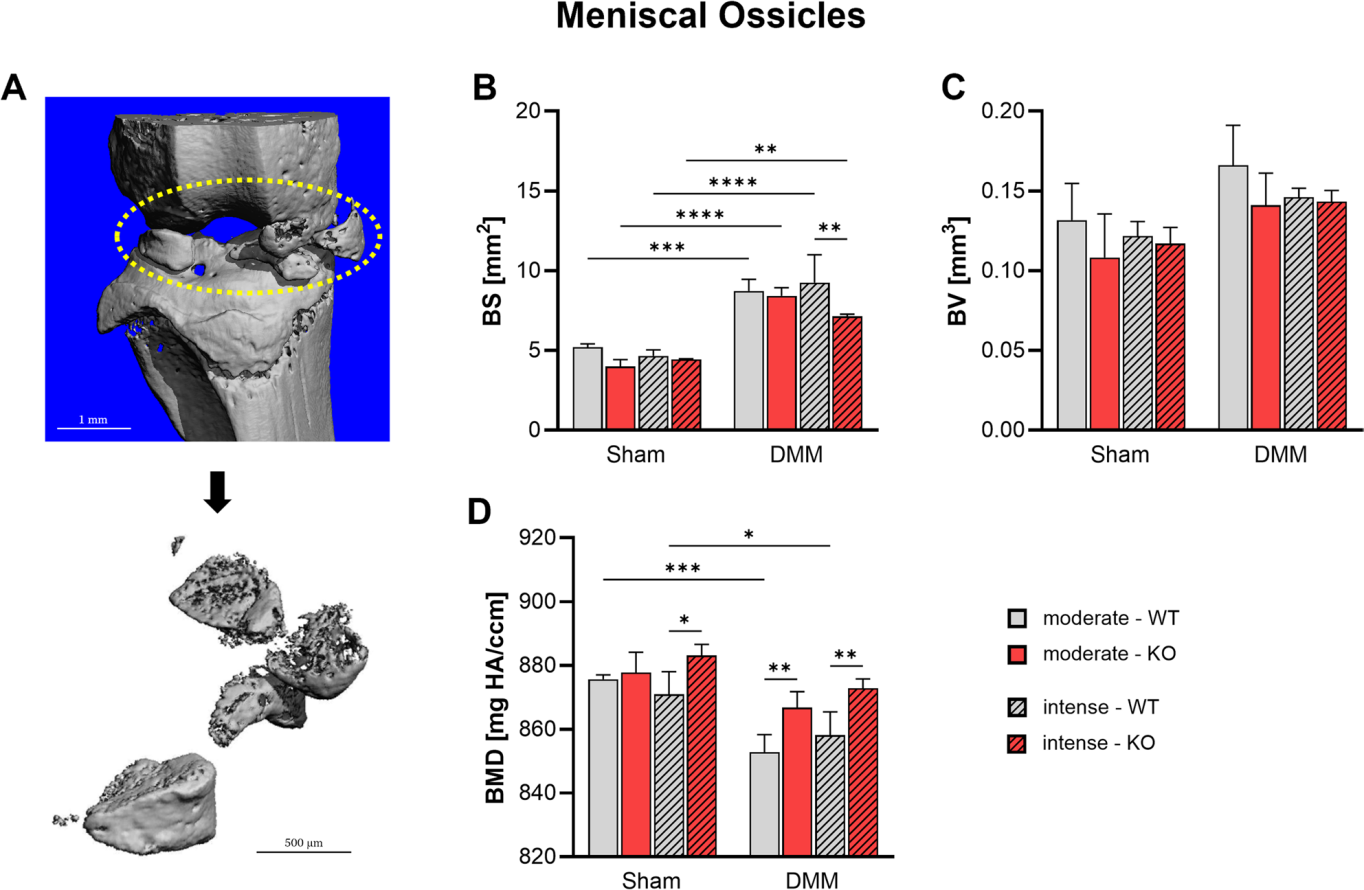
Effect of Tac1 deficiency and forced exercise on meniscal heterotopic ossification after OA-induction. Ultra-high resolution nanoCT analysis of medial and lateral meniscal ossicle formation in WT and KO mice exposed to moderate or intense exercise at 8 weeks after DMM or Sham surgery. **A** Representative image showing the region of interest (Circle). Analysis of (**B**) bone surface (BS), (**C**) bone volume (BV), and (**D**) bone mineral density (BMD). Statistical analysis using Two-Way ANOVA and Tukey’s multiple comparisons test. * *p* < 0.05, ** *p* < 0.01, *** *p* < 0.001, **** *p* < 0.0001. *N* = 3

High-resolution nanoCT showed ectopic bone formation within the meniscus 8 weeks post-surgery (Fig. 4, Suppl. Figure 5). Bone surface (BS) area increased markedly in all groups after DMM (Fig. 4B). However, KO mice subjected to intense exercise displayed a significantly smaller BS than WT under the same conditions. Bone volume (BV) showed a modest, non-significant increase in all DMM groups, consistent with a trend toward OA-associated ossification (Fig. 4C). At the same time bone mineral density (BMD) was found to be significantly reduced in WT mice following DMM. Here, KO mice exhibited significantly higher BMD after DMM compared to WT their counterparts. Notably, intense exercise increased BMD in Tac1 KO mice independent of OA in the Sham group (Fig. 4D).

### Osteophyte formation is induced by DMM

**Fig. 5:**
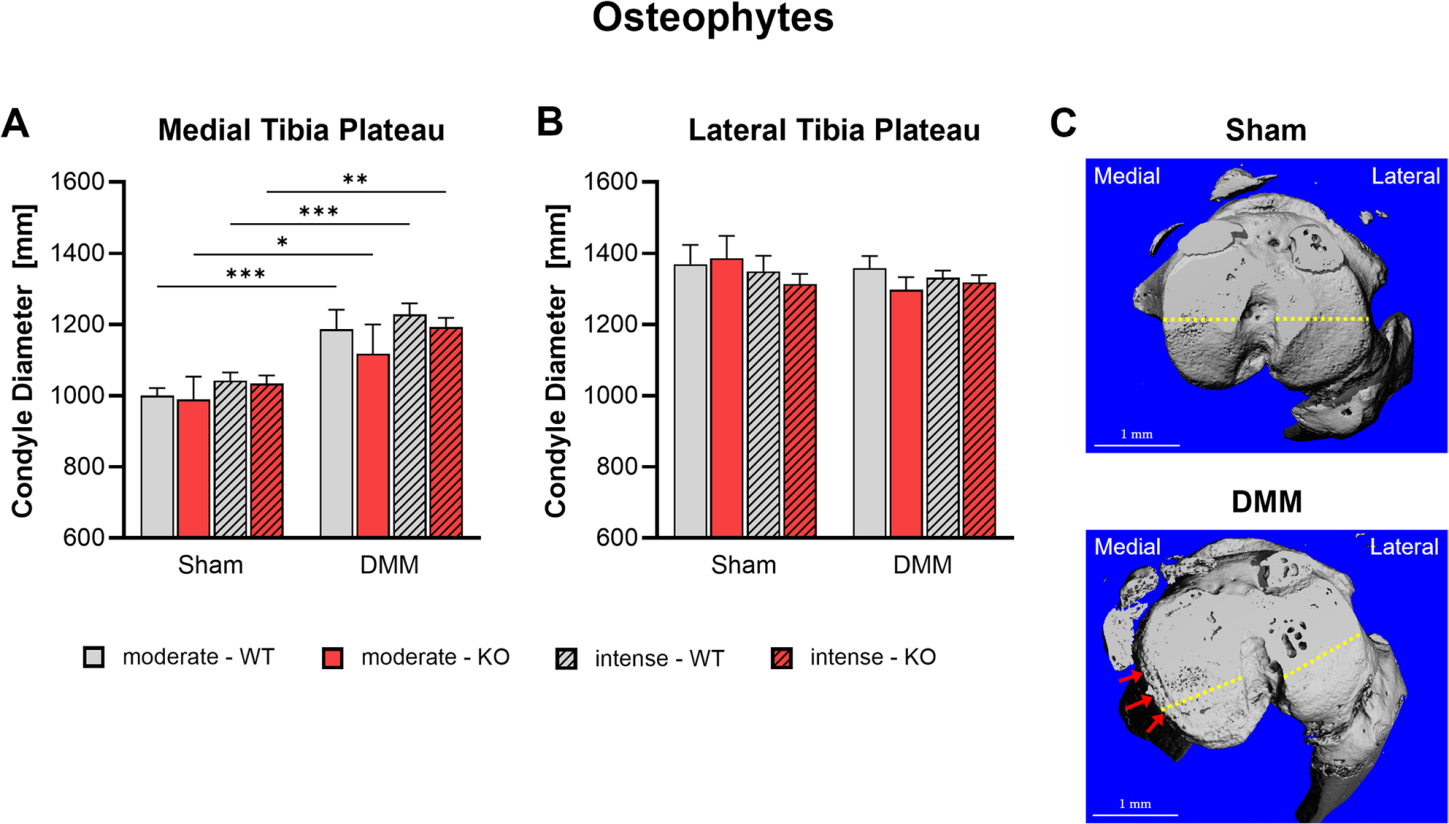
Effect of Tac1 deficiency and forced exercise on osteophyte formation after OA-induction. Ultra-high resolution nanoCT analysis of the medial (**A**) and lateral (**B**) tibia plateau diameter –correlated to osteophyte formation- in WT and KO mice exposed to moderate or intense exercise at 8 weeks after DMM or Sham surgery. Representative images of Sham and DMM mice (**C**) show location of measurement in yellow. Red arrows indicate area of osteophyte formation. Statistical analysis using Two-Way ANOVA and Tukey’s multiple comparisons test. * *p* < 0.05, ** *p* < 0.01, *** *p* < 0.001, **** *p* < 0.0001. *N* = 3

Osteophyte development was assessed via nanoCT by measuring medial and lateral tibial plateau diameters 8 weeks post-surgery (Fig. 5). DMM induced significant increases in medial tibia plateau diameters across all groups correlating with osteophyte formation (Fig. 5A, C). Tac1-deficient mice exhibited slightly reduced diameter and thus osteophyte size compared to WT mice after DMM, though differences were not statistically significant. Exercise intensity did not influence osteophyte formation in either genotype. The lateral tibial plateau diameter remained unaffected by DMM, genotype, or exercise regimen, indicating site-specific and thus load dependent osteophyte development (Fig. 5B, C).

### Tac1 deficiency and exercise modulate subchondral bone remodeling in the medial tibia after OA induction

**Fig. 6:**
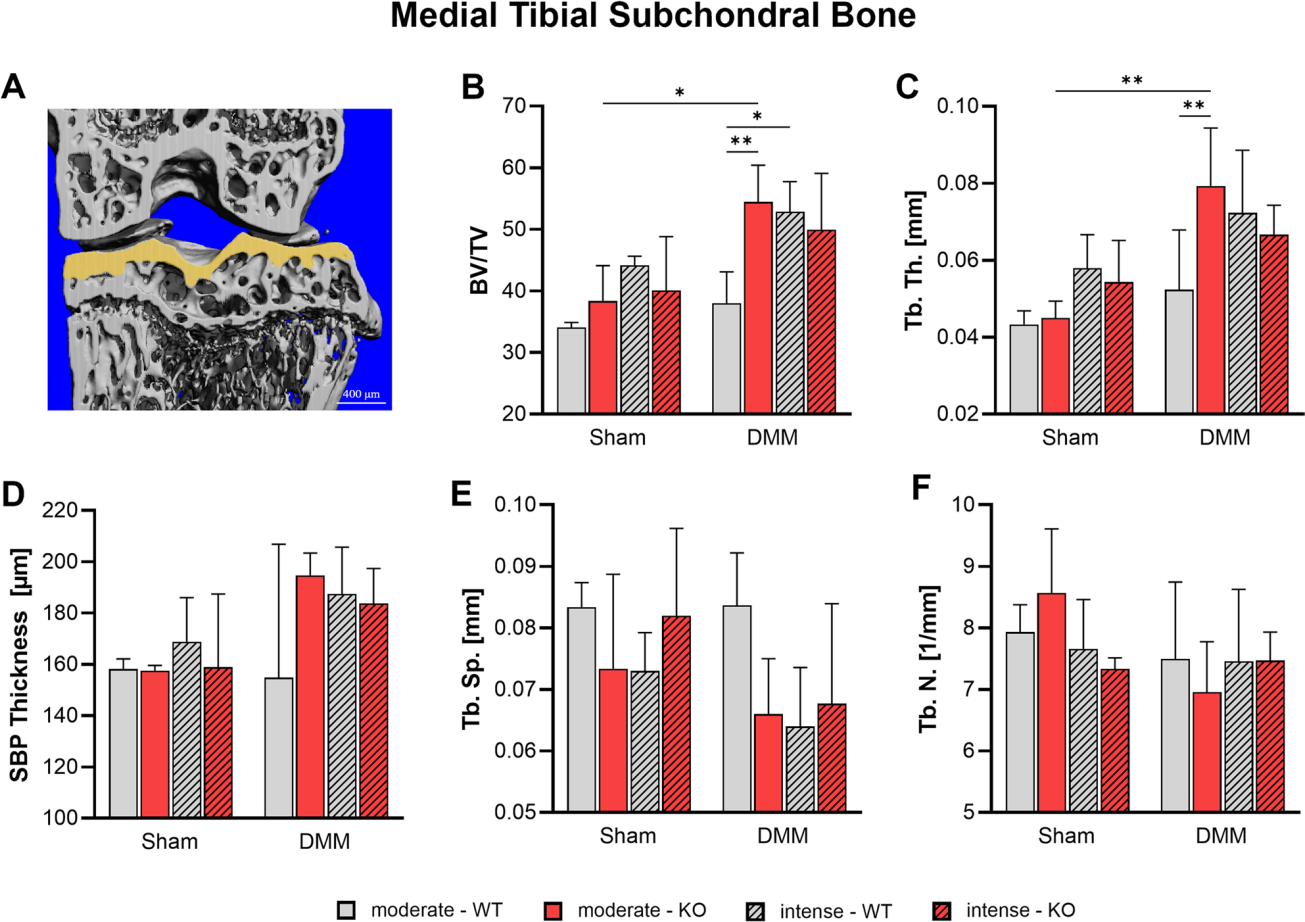
Effect of Tac1 deficiency and forced exercise on subchondral bone morphology after OA-induction. Ultra-high resolution nanoCT analysis of the subchondral bone of the medial tibia in WT and KO mice exposed to moderate or intense exercise at 8 weeks after DMM or Sham surgery. **A** Representative image with region of interest marked in yellow. Analysis of (**B**) bone volume to total volume ratio (BV/TV), (**C**) trabecular thickness (Tb. Th.), (**D**) subchondral bone plate (SBP) thickness, (**E**) trabecular separation (Tb. Sp.), and (**F**) trabecular number (Tb. N.). Statistical analysis using Two-Way ANOVA and Tukey’s multiple comparisons test. * *p* < 0.05, ** *p* < 0.01, *** *p* < 0.001. *N* = 3

Changes in medial tibial subchondral bone morphology were assessed 8 weeks after DMM surgery using nanoCT analysis (Fig. 6, Suppl. Figure 6). Bone volume fraction (BV/TV) and trabecular thickness (Tb. Th.) were increased following DMM, with the most pronounced and statistically significant changes observed in KO mice compared to the WT DMM group after moderate exercise (Fig. 6B, C). In contrast, DMM WT mice subjected to moderate exercise were protected from these alterations, showing BV/TV and Tb. Th. values comparable to Sham controls. Subchondral bone plate (SBP) thickness exhibited similar trends of increase pattern following DMM, although differences did not reach statistical significance (Fig. 6D). Trabecular separation (Tb. Sp.) was reduced by trend after DMM in all exercise groups, indicating trabecular compaction; however, this effect was absent in WT mice following moderate exercise, which retained Tb. Sp. values comparable to the corresponding WT Sham animals (Fig. 6E). Trabecular number (Tb. N.) remained unaffected by DMM, genotype, or exercise intensity (Fig. 6F).

### Tac1 deficiency alters metaphyseal bone properties in response to mechanical loading and OA in the medial tibia

**Fig. 7:**
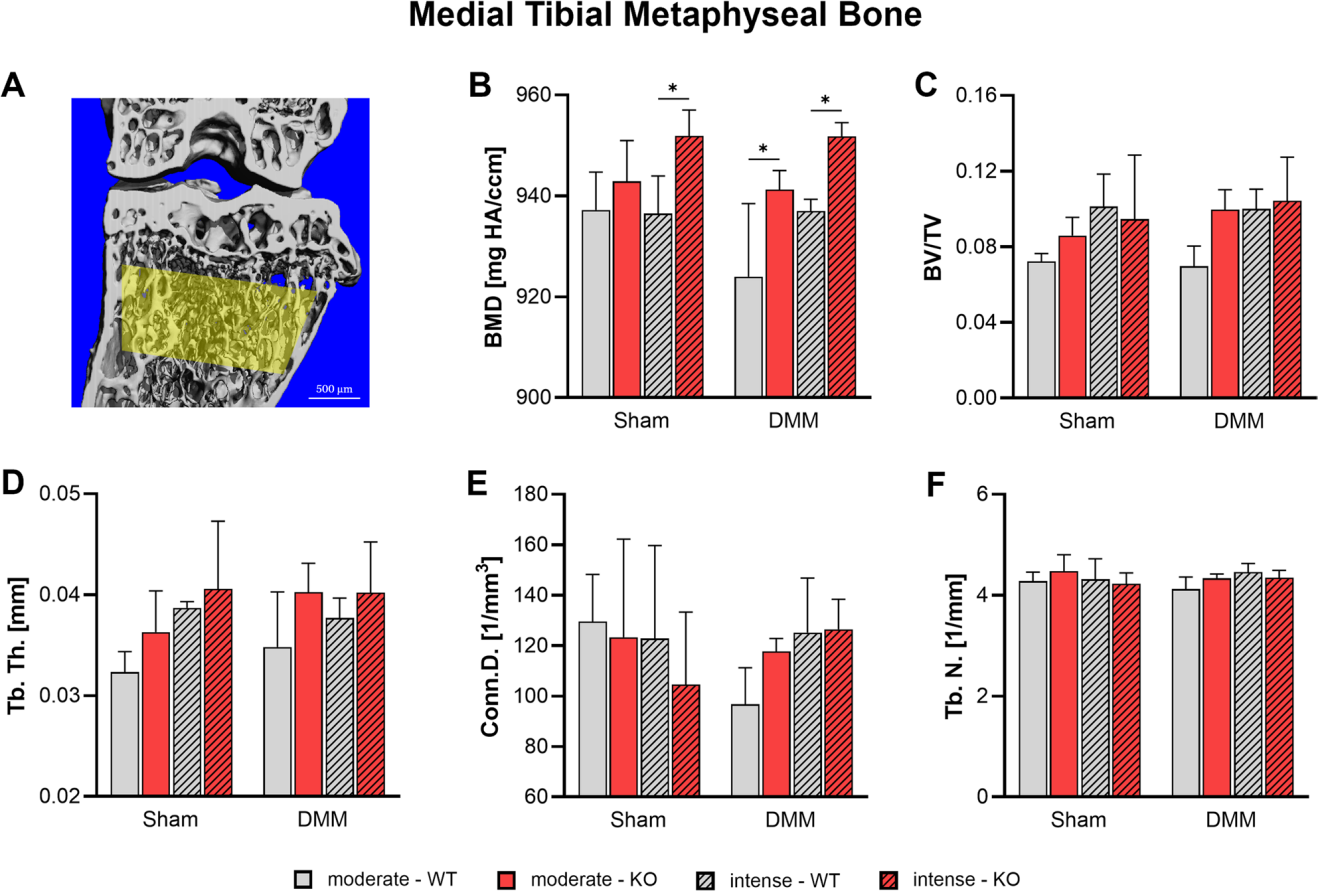
Effect of Tac1 deficiency and forced exercise on metaphyseal bone morphology after OA-induction. Ultra-high resolution nanoCT analysis of the metaphyseal bone of the medial tibia in WT and KO mice exposed to moderate or intense exercise at 8 weeks after DMM or Sham surgery. **A** Representative image with region of interest marked in yellow. Analysis of (**B**) bone mineral density (BMD), (**C**) bone volume to total volume ratio (BV/TV), (**D**) trabecular number (Tb. N.), (**E**) Connectivity Density (Conn. D.), and (**F**) trabecular thickness (Tb. Th.). Statistical analysis using Two-Way ANOVA and Tukey’s multiple comparisons test. * *p* < 0.05, ** *p* < 0.01. *N* = 3

Following the observation of subchondral bone sclerosis, nanoCT analysis was extended to the underlying trabecular metaphyseal bone region (Fig. 7, Suppl. Figure 7). Bone mineral density (BMD) was significantly increased in KO mice after intense exercise, irrespective of OA induction, while WT mice exhibited a significant reduction in BMD following DMM and moderate exercise (Fig. 7B). BV/TV and Tb. Th. were increased in both Sham and DMM KO groups relative to WT mice by trend when subjected to moderate exercise (Fig. 7C, D). Conn. D. showed a trend toward reduction in KO Sham mice following intense exercise and in WT mice after DMM with moderate exercise (Fig. 7E). Tb. N. remained unaffected by DMM, Tac1 deficiency, or exercise intensity (Fig. 7F).

### Tac1 deficiency alters serum inflammatory marker levels in response to mechanical loading

**Fig. 8:**
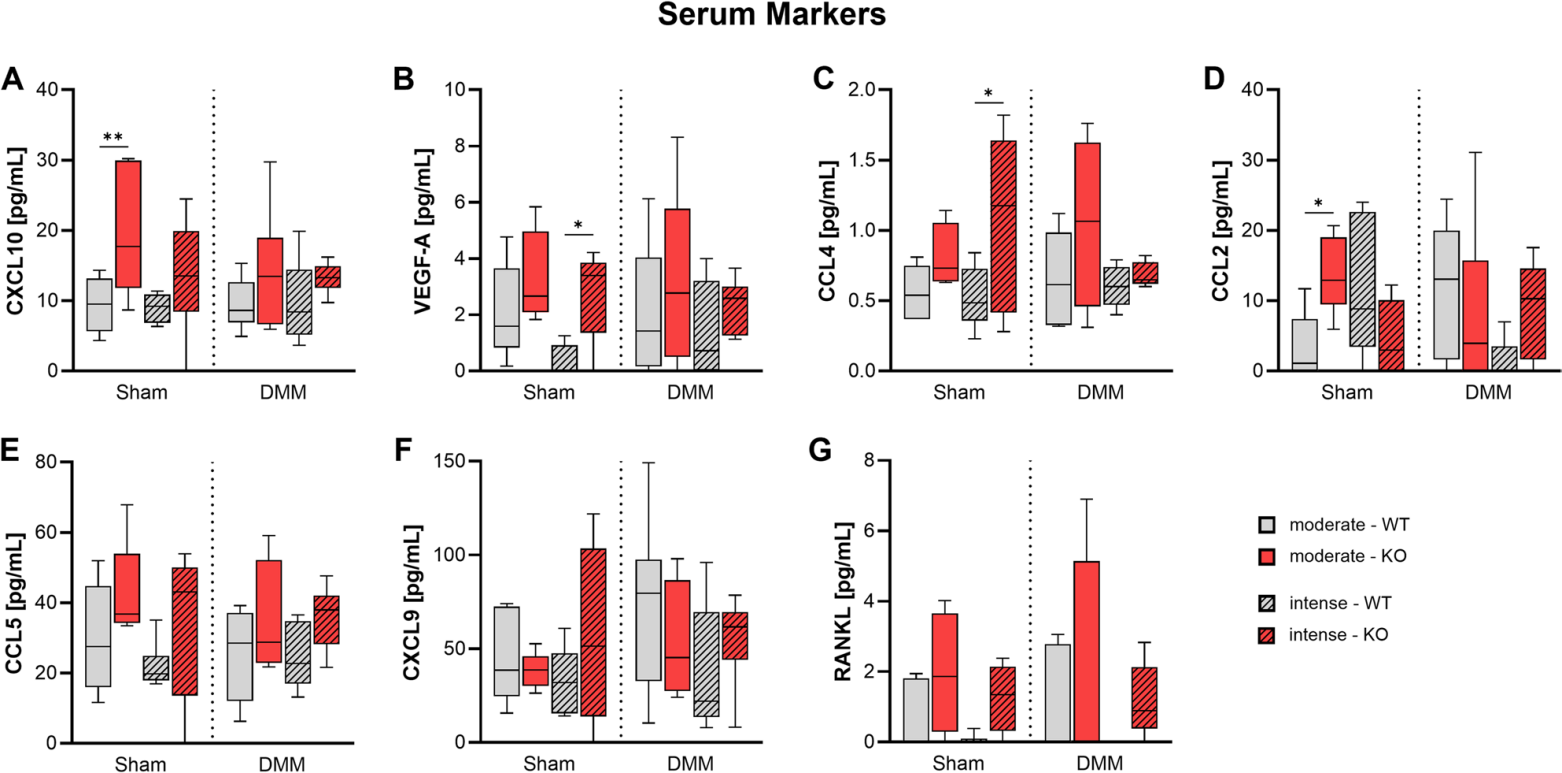
OA-associated serum markers in Tac1 deficient mice after OA-induction and forced exercise. Selected serum marker concentrations of WT and KO mice exposed to moderate or intense exercise at 8 weeks after Sham or DMM surgery. Statistical analysis using Two-Way ANOVA and Tukey’s multiple comparisons test. * *p* < 0.05, ** *p* < 0.01, *** *p* < 0.001, **** *p* < 0.0001. *N* = 6

Serum concentrations of proinflammatory and OA-associated markers were measured 8 weeks after surgery (Fig. 8). CXCL10 levels were significantly elevated in KO mice compared to WT following Sham surgery under moderate exercise (Fig. 8A). Similarly, VEGF-A and CCL4 concentrations were significantly increased in KO mice after Sham surgery following intense exercise (Fig. 8B, C). CCL2 levels were also significantly higher in KO versus WT mice in the Sham group exposed to moderate loading (Fig. 8D). No significant differences of these markers were observed in the DMM groups and for the remaining markers (Fig. 8E-G). Even though an induction of all analyzed markers by trend can be observed in the DMM KO groups after intense exercise compared to the related DMM WT groups.

## Discussion

Mechanical loading through exercise exerts both beneficial and harmful effects in the context of OA, depending on its intensity and timing. In rodent models of OA induced by DMM, anterior cruciate ligament transection (ACL-T), or high-fat diet, moderate levels of exercise have been shown to protect against cartilage degradation and slow the progression of subchondral bone sclerosis [14, 22–25]. In contrast, high-intensity physical activity following OA induction has been associated with exacerbated joint damage and increased severity of OA-related lesions [16, 23, 26]. Notably, studies have demonstrated that treadmill running alone, in the absence of surgical or dietary interventions, can initiate OA-like changes, indicating that excessive mechanical stress may itself trigger joint degeneration [15, 27, 28]. While the precise molecular pathways underlying these effects remain unclear, the sensory neuropeptide SP has emerged as a promising candidate for further investigation. SP has been implicated in the regulation of chondrocyte and bone cell responses to mechanical stimuli and has also been associated with structural alterations in bone during OA development [3, 7–9]. Therefore, the present study aimed to examine how the absence of SP, in combination with varying exercise intensities, influences the progression of OA.

As anticipated, destabilization of the medial meniscus (DMM) surgery generally led to medial tibial and femoral cartilage degeneration after six weeks of either moderate or intense treadmill exercise. A notable exception were SP KO mice subjected to intense exercise after DMM surgery and which seemed protected from cartilage degradation and showed OARSI scores similar to those of their Sham counterparts. Our moderate exercise protocol on the other hand did not confer any protective effect on the cartilage. This contrasts with studies reporting cartilage preservation following moderate exercise, which often employed lower treadmill speeds, flat (no incline) surfaces that reduce hindlimb stress, or allowed for longer recovery periods between surgery and exercise initiation. Additionally, Iijima et al. conducted their experiments in rats, where the same exercise conditions may have imposed relatively lower mechanical stress due to the animals’ larger size [14, 23, 24]. Taken together, these differences suggest that the parameters of our moderate exercise regimen imposed a higher biomechanical load, eliciting cartilage degeneration comparable to that observed with intense exercise protocols in rat models.

AFM is a powerful tool to detect microscale changes in articular cartilage that occur before structural damage is detected with macroscale technologies like histology. Its high sensitivity and spatial resolution is capable of detecting changes in tissue stiffness at the nano- and microscale [29, 30]. For instance, alterations in the composition of the extracellular matrix (ECM), such as the decrease in aggrecan content and changes in collagen concentration, precede the structural changes scored by systems like OARSI [29]. AFM is uniquely suited to detect these precursors to damage, as it measures the mechanical properties related to both the proteoglycan moiety and the collagen fibrillar network (represented by bimodal stiffness distributions) [15, 28]. In line with our histological findings, SP KO mice subjected to DMM surgery and intense treadmill exercise maintained matrix stiffness values—mediated by proteoglycans and collagen—in the deep zone of the articular cartilage at levels comparable to Sham-operated controls. In contrast, WT mice exposed to the same intense exercise displayed the highest deep zone cartilage Young’s modulus values observed in this setting. Notably, moderate exercise did not exert a protective effect in SP KO mice. This was also true for our previous work, which showed increased cartilage stiffness in SP-deficient mice following OA induction in the absence of forced exercise [3]. Alterations in the composition and structural organization of the extracellular matrix (ECM), particularly involving large aggregating proteoglycans and fibrillar collagens, are well-established features of OA progression [31, 32]. During early OA, bulk cartilage stiffness typically decreases at the tissue level, while nanoscale stiffness—particularly of collagen fibrils—often increases, reflecting microstructural changes within the ECM [33, 34]. Our data shows that SP deficiency appears to mitigate these ECM alterations, as proteoglycan and collagen fiber stiffening were absent following OA induction and intense mechanical loading. SP plays a crucial role in cartilage health by contributing to mechanotransduction via its receptor NK1R [10, 35]. Millward-Sadler et al. [9] demonstrated that human articular chondrocytes express SP, its precursor pre-protachykinin, and NK1R both in vivo and in vitro. Exposure to SP or mechanical stimulation induced membrane hyperpolarization in cultured chondrocytes, an effect blocked by NK1R antagonists, indicating that SP mediates mechanosensory responses in chondrocytes through autocrine and/or paracrine signaling [9]. Furthermore, Karaha et al. found increased SP expression in articular chondrocytes, cartilage matrix, and synovial membrane under intense exercise conditions, suggesting a role for SP in regulating cartilage metabolism and joint homeostasis in response to mechanical load [36]. These findings highlight the regulatory function of SP in cartilage ECM remodeling under mechanical stress and indicate that this response is distinct between OA and healthy chondrocytes.

We also observed that SP deficiency, in combination with mechanical stress, led to structural remodeling of meniscal tissue. While DMM generally induced heterotopic ossification leading to the formation of ossicles, the absence of SP further promoted mineralization of these ossicles, independent of exercise intensity. Interestingly, intense exercise increased BMD of these meniscal ossicles in SP knockout mice also after Sham surgery suggesting an OA independent effect of SP deficiency. These effects were largely absent in our previous study without exercise, where DMM slightly decreased BMD but with SP deficiency not having significant influence [3]. These findings suggest that mechanical loading, even at moderate levels, may interact with SP signaling to modulate ectopic ossification in meniscal tissue.

Growing evidence suggests that changes in subchondral bone architecture play both direct and indirect roles in driving cartilage degradation and joint pain [37]. At the same time, physical activity has been reported to influence the microstructure of subchondral and metaphyseal trabecular bone [14, 38].

Subchondral bone sclerosis is a well-recognized consequence of DMM-induced OA [3, 14]. In our study, however, WT mice subjected to DMM followed by moderate treadmill exercise did not exhibit signs of subchondral bone sclerosis. Their bone architecture remained comparable to that of the Sham-operated controls, suggesting that moderate mechanical loading may help preserve subchondral bone integrity after joint injury. While SP deficiency and exercise intensity had little impact on bone structure in Sham-operated mice, the response was markedly different in the DMM group.

Following OA induction, SP KO mice, as well as WT mice exposed to intense exercise—or a combination of both factors—developed pronounced subchondral bone sclerosis. These structural changes included increased BV/TV, thickening of trabeculae and the SBP, and thereof reduced trabecular separation. Sclerotic changes, marked by significantly elevated BMD, were also observed in the underlying metaphyseal bone—but exclusively in SP KO mice following DMM and intense exercise. Notably, similar alterations in bone microarchitecture were previously observed in SP KO mice even in the absence of exercise, indicating a baseline dysregulation of bone remodeling linked to SP deficiency [3]. Supporting this, Niedermair et al. showed that SP deficiency reduces bone resorption by lowering the number of bone marrow precursor cells and osteoclasts in vitro [39]. Complementary findings by Takaaki et al. demonstrated that SP released from peripheral sensory nerves promotes osteoclastogenesis through upregulation of RANKL and downregulation of OPG in synovial fibroblasts, contributing to pathological bone turnover [40]. Consistent with our results, Li et al. reported that high-intensity treadmill running in rats led to increased SBP thickness, elevated BMD, and denser, plate-like trabecular structures—features also observed in our SP-deficient and intensely exercised mice [38]. These findings underscore the interplay between SP signaling and mechanical loading in shaping subchondral bone response and suggest that a physiological SP dose may play a protective role in regulating bone remodeling under OA conditions.

Early OA is marked by activation of both innate and adaptive immune responses. Even before clinical symptoms appear, joint inflammation alters levels of inflammatory and OA-related biomarkers in synovial fluid, serum, and urine [41, 42]. SP, released by sensory nerve fibers and immune cells such as macrophages, lymphocytes, and dendritic cells, is recognized as a neuro-inflammatory pro-nociceptive mediator, though its specific role in OA-related inflammation remains unclear [43, 44]. Unexpectedly, our study showed that SP KO mice had elevated serum levels of several proinflammatory and OA-associated cytokines, with significant increases in Sham-operated animals for CXCL10, VEGF-A, CCL4, and CCL2. Following DMM surgery, however, these differences were either diminished or highly variable between mice, making interpretation difficult. This discrepancy may stem from differences between systemic and local cytokine levels, as serum biomarkers may not fully capture joint-specific inflammatory activity. Future studies could address this limitation by incorporating joint-specific analyses, for example of synovial fluid or synovial tissue. Although the increased systemic inflammation in SP KO mice did not lead to measurable cartilage degradation in our study, such effects could emerge at later times beyond our experimental timeline. Overall, our findings suggest that SP helps regulating inflammatory responses in OA, and its absence may disrupt immune signaling in ways not immediately reflected in structural joint pathology.

Our results demonstrate the dual nature of SP and underscore the complex balance required for therapeutic targeting in human OA. Targeting the SP-NK1R pathway is feasible through two opposing strategies. SP Agonism aims for anti-sclerotic and regenerative effects: the NK1R agonist septide suppressed OA progression and inhibited subchondral bone sclerosis (lower BV/TV) in DMM mice [45]. Furthermore, SP combined with specialized delivery systems (self-assembled peptides, SAP) showed regenerative and anti-inflammatory properties, improving cartilage and recruiting mesenchymal stem cells (MSCs) [46]. SP/NK1R Antagonism targets SP as a neuro-inflammatory pro-nociceptive mediator. NK1R antagonists (e.g., aprepitant) suppress inflammatory factors in synoviocytes [46, 47]. A clinically used indirect antagonist is capsaicin, which relieves pain by depleting SP from nerves via TRPV1 activation. Advancements using nanotechnology are addressing capsaicin’s historical limitations (e.g., pungency/irritation), improving its therapeutic index and safety profile for topical delivery [47, 48]. The primary translational challenge lies in avoiding disruption of SP’s crucial homeostatic functions while achieving effective pain relief. General clinical trials for NK1R antagonists have been disappointing [47]. Moreover, interventional SP-targeting protein therapeutics (e.g., SP-Saporin) face procedural challenges and risks (e.g., motor deficits), necessitating validation in transitional large animal models before human use [49]. Ultimately, successful SP-based therapies depend critically on optimizing the dose, continuity of release, and targeted delivery of conjugates [46].

It is important to acknowledge the limitations of this study and their potential influence on the interpretation of our findings. One key limitation is the exclusive use of male animals. Numerous studies have reported that female mice typically develop less severe OA symptoms following DMM surgery, making them less suitable for post-traumatic OA (PTOA) models [13, 50–52]. However, a recent systematic analysis has suggested that both male and female C57BL/6 mice exhibit comparable levels of joint damage after DMM surgery [53]. Given the higher prevalence of OA in women over the age of 50, future studies should incorporate female mice to enhance translational relevance [1]. Another limitation is the absence of littermate controls. In this study, SP KO mice were bred in-house, while WT C57BL/6J mice were acquired from Charles River Laboratories. This introduces the potential for genetic and phenotypic variability unrelated to the targeted gene deletion, thereby increasing the risk of confounding results or false-positive findings [54]. Nonetheless, this approach aligns with the 3R (Replacement, Reduction, and Refinement) principles by avoiding unnecessary breeding of mice with undesired genotypes and minimizing surplus animals. Another limitation of the study is the absence of a sedentary, non-exercise control group, which restricts the ability to distinguish exercise-specific effects from baseline OA progression. However, implementing moderate exercise as the baseline condition was intended to provide an uniform level of mechanical loading across all animals. This strategy also aimed to reduce variability between individual mice due to potential hypoactivity caused by DMM-induced joint instability or pain, thereby improving consistency between experimental groups.

## Conclusion

This study highlights the complex, context-dependent role of SP in OA progression under mechanical stress. While moderate exercise did not protect cartilage in our DMM-surgical model, SP deficiency produced distinct tissue responses, affecting cartilage stiffness, meniscal heterotopic ossification, and subchondral and metaphyseal bone remodeling. Surprisingly, SP-deficient mice subjected to intense exercise showed preserved cartilage morphology and cartilage matrix stiffness, yet also developed pronounced meniscal ossification and subchondral bone sclerosis, indicating a dual role for SP in joint protection and pathological remodeling. SP deficiency also led to elevated serum levels of proinflammatory cytokines, even without OA induction, suggesting aberrant systemic immune modulation. However, these inflammatory changes did not immediately impact cartilage integrity. Overall, our findings support SP as a central neuro-mediator at the intersection of neuroinflammation, mechanotransduction, and bone–cartilage interaction in OA.

## Supplementary Information

Supplementary Material 1: Figure S1. Impact of Tac1 deficiency and exercise intensity on cartilage degradation after OA induction. Representative images of Safranin-O stained frontal sections of paraffin embedded knee joints of WT and KO mice exposed to moderate or intense exercise. Cartilage of (A) the medial tibia plateau (MTP) and femoral condyle (MFC) as well as (B) the lateral tibia plateau (LTP) and femoral condyle (LFC) were graded 4 and 8 weeks after Sham or DMM surgery. MM/LM = medial/lateral meniscus.

Supplementary Material 2: Figure S2. Impact of Tac1 deficiency and exercise intensity on lateral cartilage degradation after OA induction. Cartilage was evaluated for grades of destruction according to the OARSI guidelines for murine OA. Cartilage of the right knee joints of WT and KO mice exposed to moderate or intense exercise were graded 4 weeks (A) and 8 weeks (B) after Sham or DMM surgery. Means of the sum maximal OARSI scores of the lateral tibial and femoral cartilage were compared. Statistical analysis using Kruskal-Wallis and Dunn’s test for multiple comparisons. * p<0.05, ** p<0.01. N=6-10.

Supplementary Material 3: Figure S3. Atomic force microscopy-based analysis of the superficial cartilage matrix stiffness in Tac1 deficient mice after OA-induction and forced exercise. Analysis of articular cartilage surface properties of the right knee joint of WT and KO mice exposed to moderate and intense exercise at 8 weeks after DMM or Sham surgery. Histograms of Young’s modulus (stiffness) distributions of the superficial zone cartilage matrix. The black line in each histogram represents a fit to the data using a linear combination of two Gaussian distributions. The dashed black lines show the individual Gaussian distributions representing the proteoglycan (left) and the collagen (right) Young's moduli, respectively, as described in detail in the methods section. N=3.

Supplementary Material 4: Figure S4. Atomic force microscopy-based analysis of the middle zone cartilage matrix stiffness in Tac1 deficient mice after OA-induction and forced exercise. Analysis of articular cartilage surface properties of the right knee joint of WT and KO mice exposed to moderate and intense exercise at 8 weeks after DMM or Sham surgery. Histograms of Young’s modulus (stiffness) distributions of the middle zone cartilage matrix. The black line in each histogram represents a fit to the data using a linear combination of two Gaussian distributions. The dashed black lines show the individual Gaussian distributions representing the proteoglycan (left) and the collagen (right) Young's moduli, respectively, as described in detail in the methods section. N=3.

Supplementary Material 5: Figure S5. Effect of Tac1 deficiency and forced exercise on osteophyte formation after OA-induction. Representative images of ultra-high resolution nanoCT analysis of medial and lateral meniscal ossicle formation in WT and KO mice exposed to moderate or intense exercise at 8 weeks after DMM or Sham surgery.

Supplementary Material 6: Figure S6. Effect of Tac1 deficiency and forced exercise on subchondral bone morphology after OA-induction. Representative images of ultra-high resolution nanoCT analysis of the subchondral bone of the medial tibia in WT and KO mice exposed to moderate or intense exercise at 8 weeks after DMM or Sham surgery.

Supplementary Material 7: Figure S7. Effect of Tac1 deficiency and forced exercise on metaphyseal bone morphology after OA-induction. Representative images of ultra-high resolution nanoCT analysis of the metaphyseal bone of the medial tibia in WT and KO mice exposed to moderate or intense exercise at 8 weeks after DMM or Sham surgery.

## Data Availability

The datasets used and/or analyzed during the current study are available from the corresponding author on reasonable request.
