# Supplementary figures and images for: Exercise-Dependent effects of substance P deficiency on joint degeneration and inflammation in a surgical mouse model of osteoarthritis
Source: Arthritis Res Ther. 2025 Dec 4;27:224. doi: 10.1186/s13075-025-03693-7 (PMC12699830; doi:10.1186/s13075-025-03693-7)

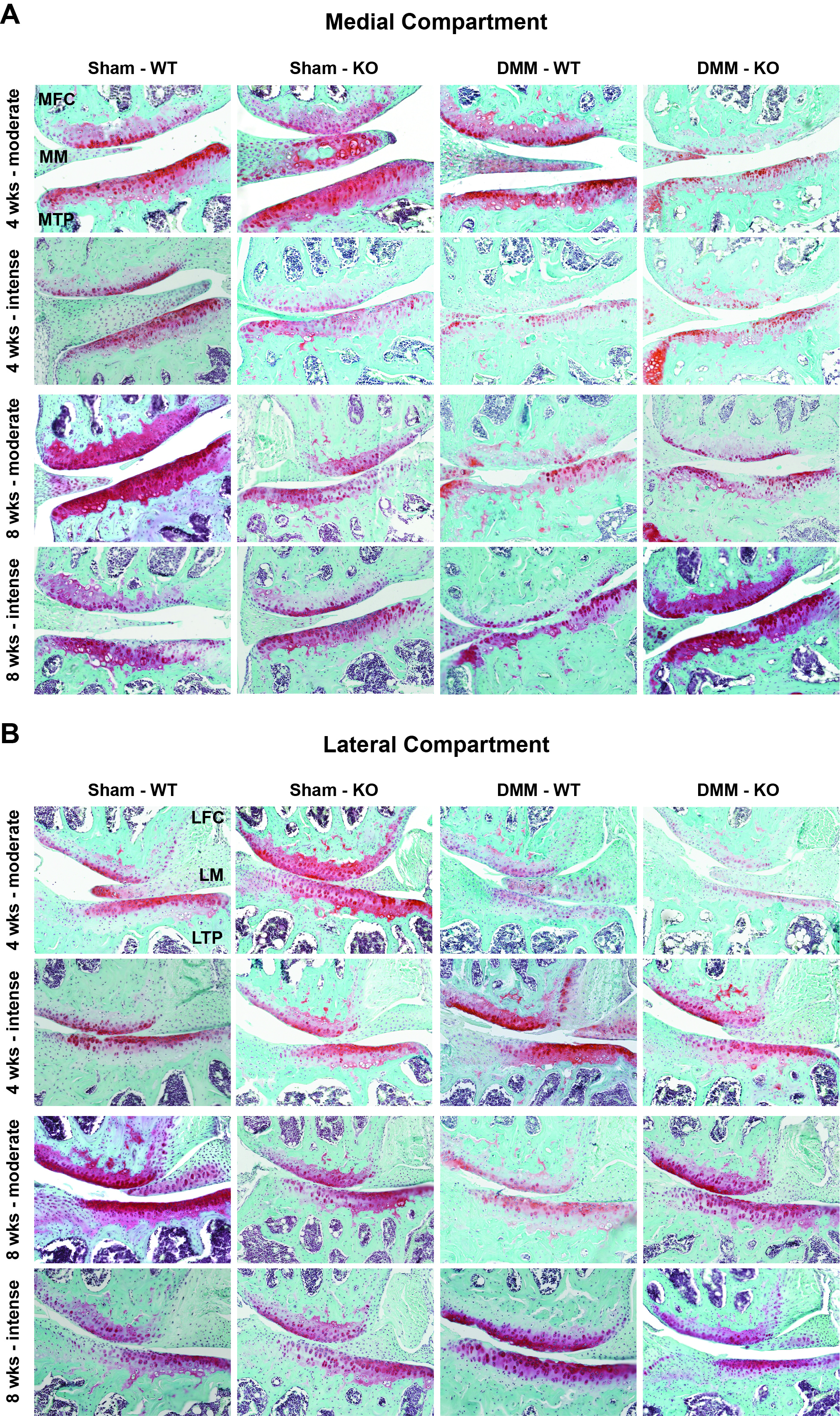

Supplement: Supplementary file 1 — Supplementary Material 1: Figure S1. Impact of Tac1 deficiency and exercise intensity on cartilage degradation after OA induction. Representative images of Safranin-O stained frontal sections of paraffin embedded knee joints of WT and KO mice exposed to moderate or intense exercise. Cartilage of (A) the medial tibia plateau (MTP) and femoral condyle (MFC) as well as (B) the lateral tibia plateau (LTP) and femoral condyle (LFC) were graded 4 and 8 weeks after Sham or DMM surgery. MM/LM = medial/lateral meniscus. [file 13075_2025_3693_MOESM1_ESM.jpg]

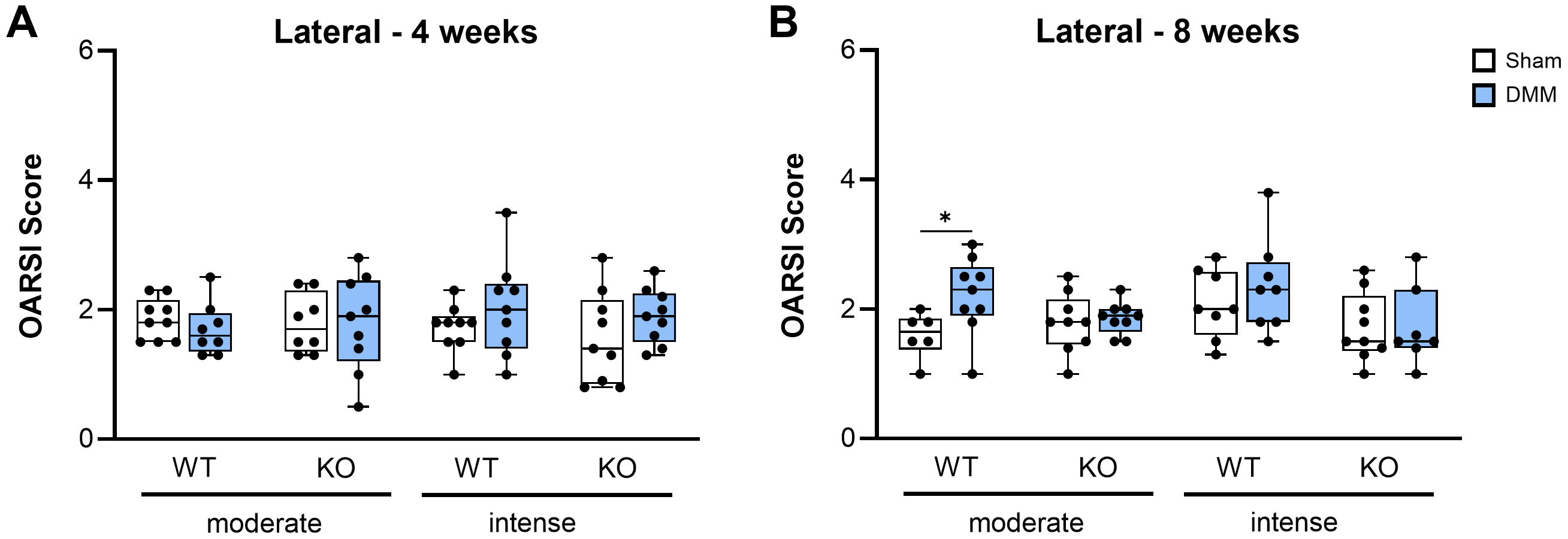

Supplement: Supplementary file 2 — Supplementary Material 2: Figure S2. Impact of Tac1 deficiency and exercise intensity on lateral cartilage degradation after OA induction. Cartilage was evaluated for grades of destruction according to the OARSI guidelines for murine OA. Cartilage of the right knee joints of WT and KO mice exposed to moderate or intense exercise were graded 4 weeks (A) and 8 weeks (B) after Sham or DMM surgery. Means of the sum maximal OARSI scores of the lateral tibial and femoral cartilage were compared. Statistical analysis using Kruskal-Wallis and Dunn’s test for multiple comparisons. * p<0.05, ** p<0.01. N=6-10. [file 13075_2025_3693_MOESM2_ESM.png]

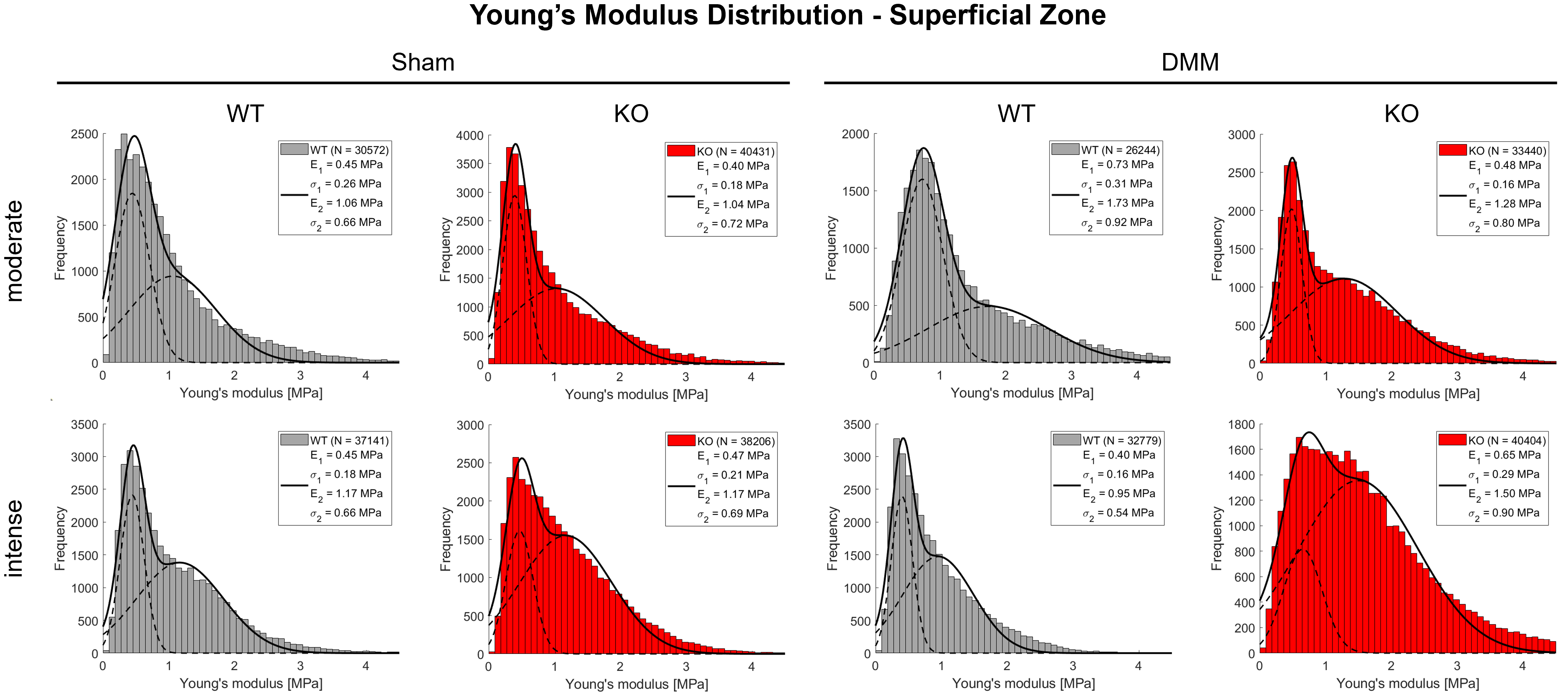

Supplement: Supplementary file 3 — Supplementary Material 3: Figure S3. Atomic force microscopy-based analysis of the superficial cartilage matrix stiffness in Tac1 deficient mice after OA-induction and forced exercise. Analysis of articular cartilage surface properties of the right knee joint of WT and KO mice exposed to moderate and intense exercise at 8 weeks after DMM or Sham surgery. Histograms of Young’s modulus (stiffness) distributions of the superficial zone cartilage matrix. The black line in each histogram represents a fit to the data using a linear combination of two Gaussian distributions. The dashed black lines show the individual Gaussian distributions representing the proteoglycan (left) and the collagen (right) Young's moduli, respectively, as described in detail in the methods section. N=3. [file 13075_2025_3693_MOESM3_ESM.png]

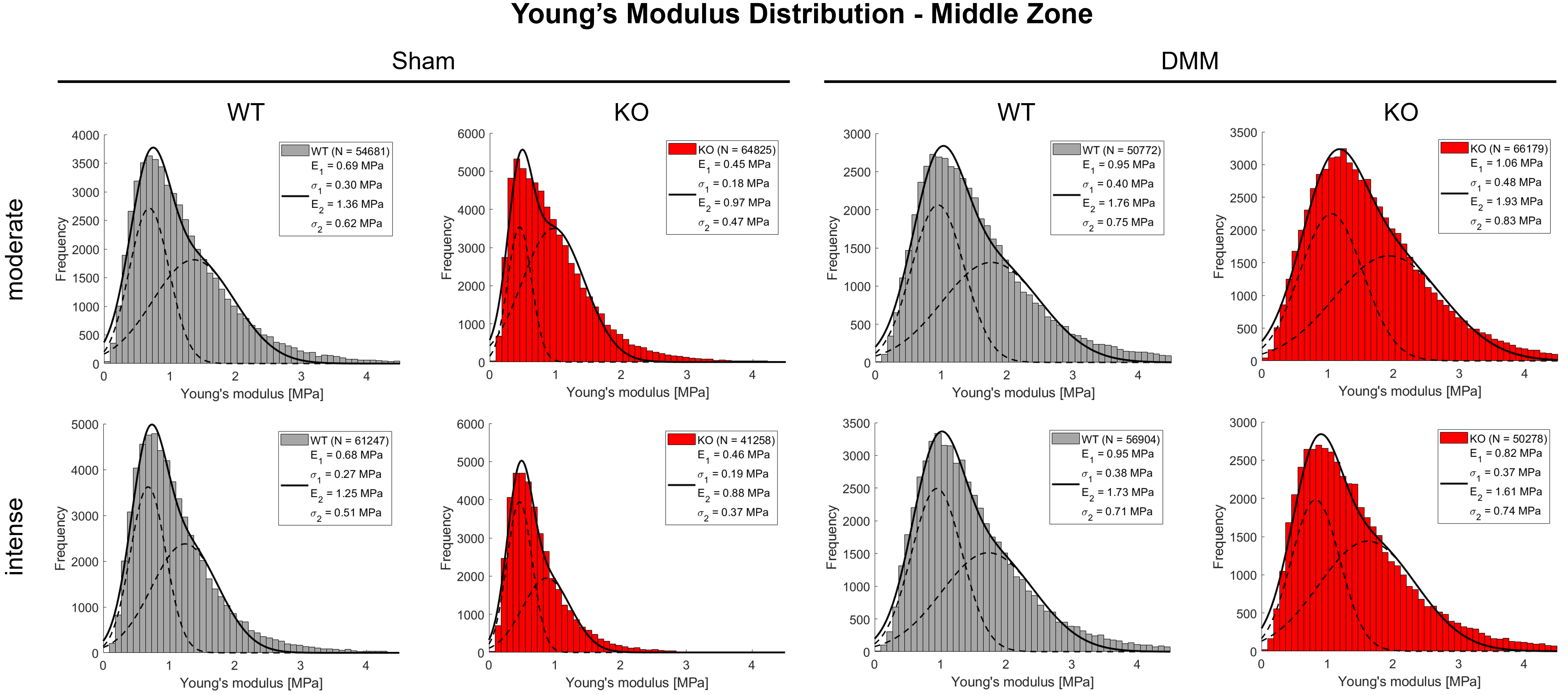

Supplement: Supplementary file 4 — Supplementary Material 4: Figure S4. Atomic force microscopy-based analysis of the middle zone cartilage matrix stiffness in Tac1 deficient mice after OA-induction and forced exercise. Analysis of articular cartilage surface properties of the right knee joint of WT and KO mice exposed to moderate and intense exercise at 8 weeks after DMM or Sham surgery. Histograms of Young’s modulus (stiffness) distributions of the middle zone cartilage matrix. The black line in each histogram represents a fit to the data using a linear combination of two Gaussian distributions. The dashed black lines show the individual Gaussian distributions representing the proteoglycan (left) and the collagen (right) Young's moduli, respectively, as described in detail in the methods section. N=3. [file 13075_2025_3693_MOESM4_ESM.png]

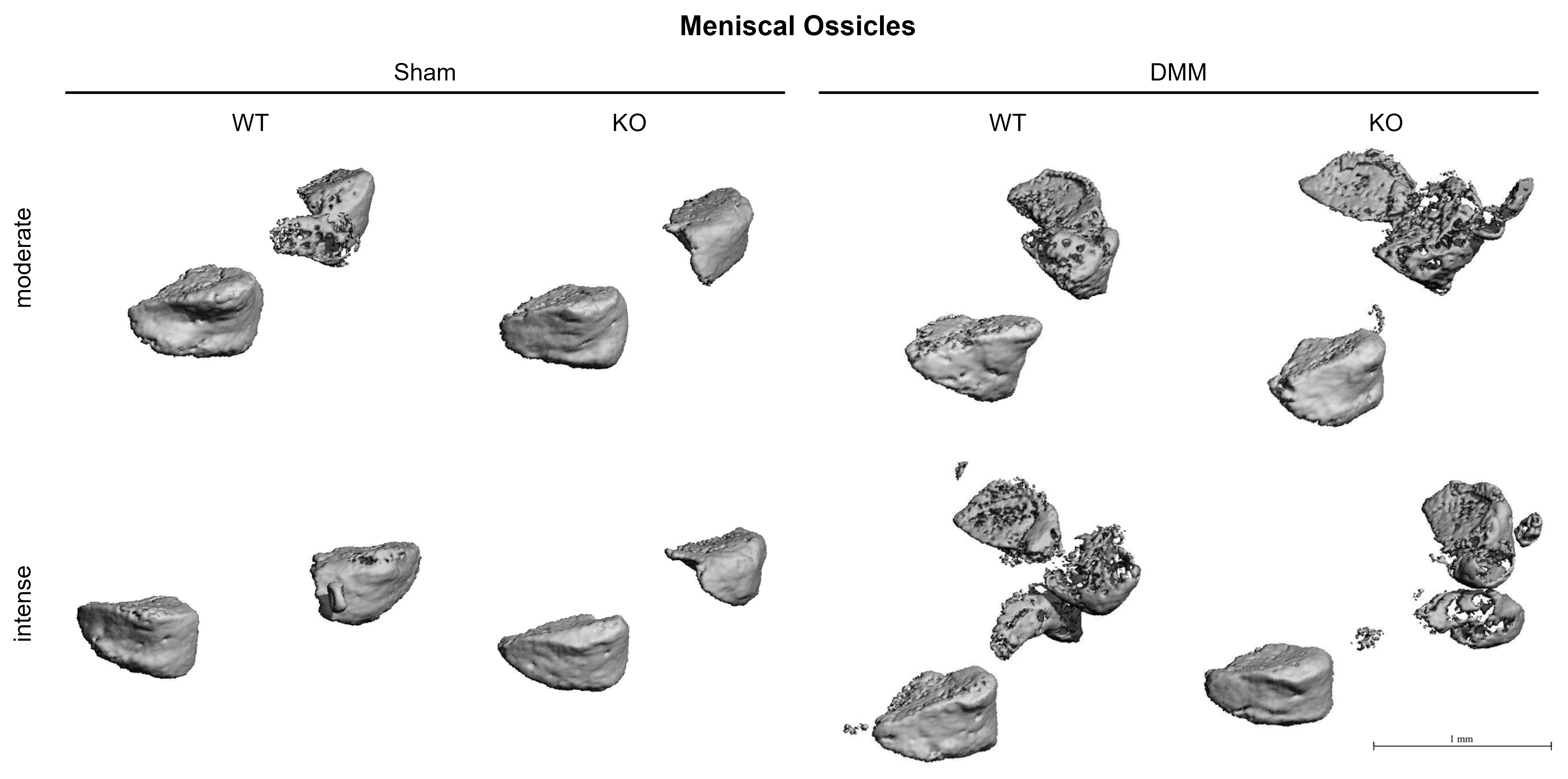

Supplement: Supplementary file 5 — Supplementary Material 5: Figure S5. Effect of Tac1 deficiency and forced exercise on osteophyte formation after OA-induction. Representative images of ultra-high resolution nanoCT analysis of medial and lateral meniscal ossicle formation in WT and KO mice exposed to moderate or intense exercise at 8 weeks after DMM or Sham surgery. [file 13075_2025_3693_MOESM5_ESM.png]

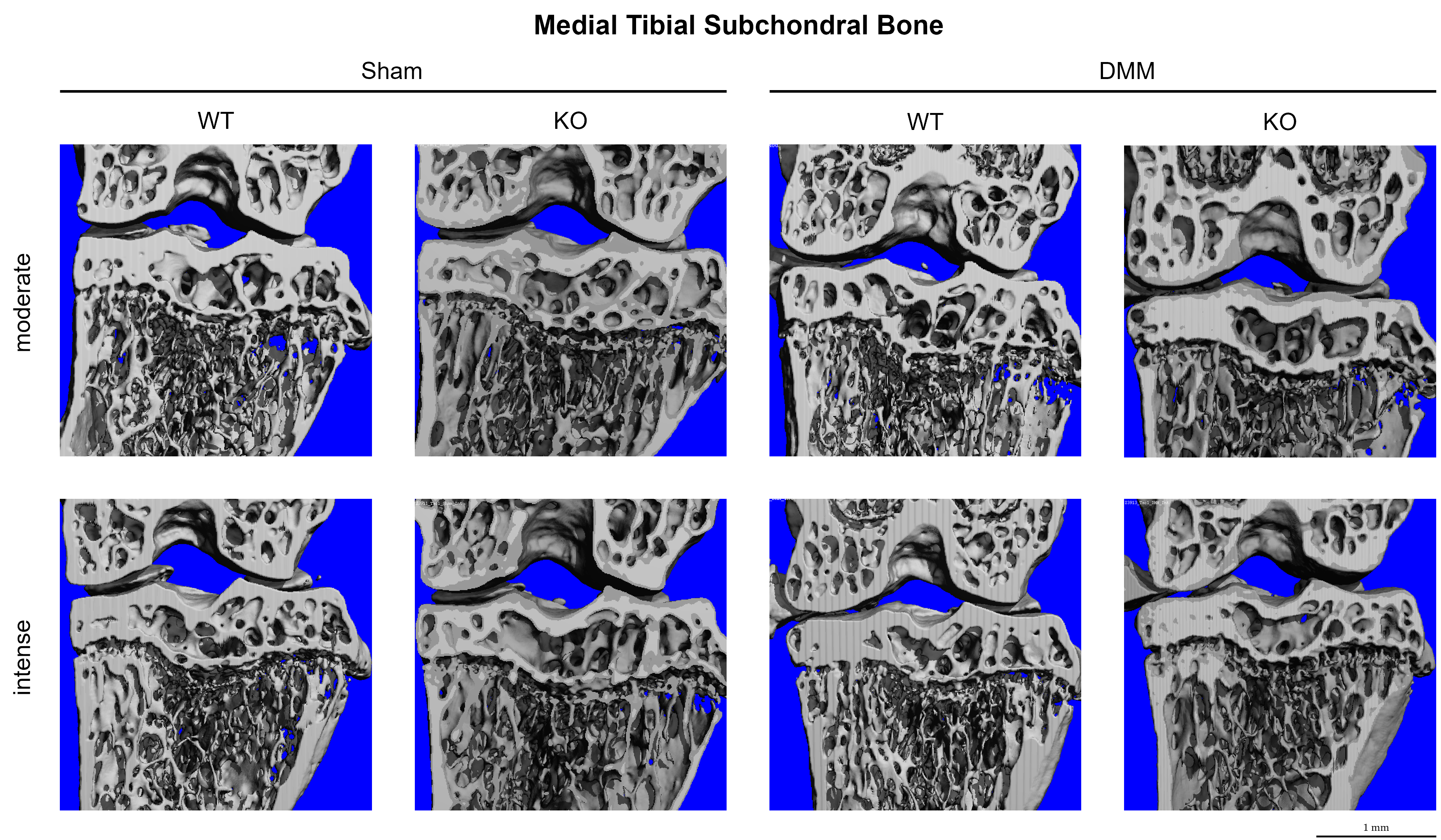

Supplement: Supplementary file 6 — Supplementary Material 6: Figure S6. Effect of Tac1 deficiency and forced exercise on subchondral bone morphology after OA-induction. Representative images of ultra-high resolution nanoCT analysis of the subchondral bone of the medial tibia in WT and KO mice exposed to moderate or intense exercise at 8 weeks after DMM or Sham surgery. [file 13075_2025_3693_MOESM6_ESM.png]

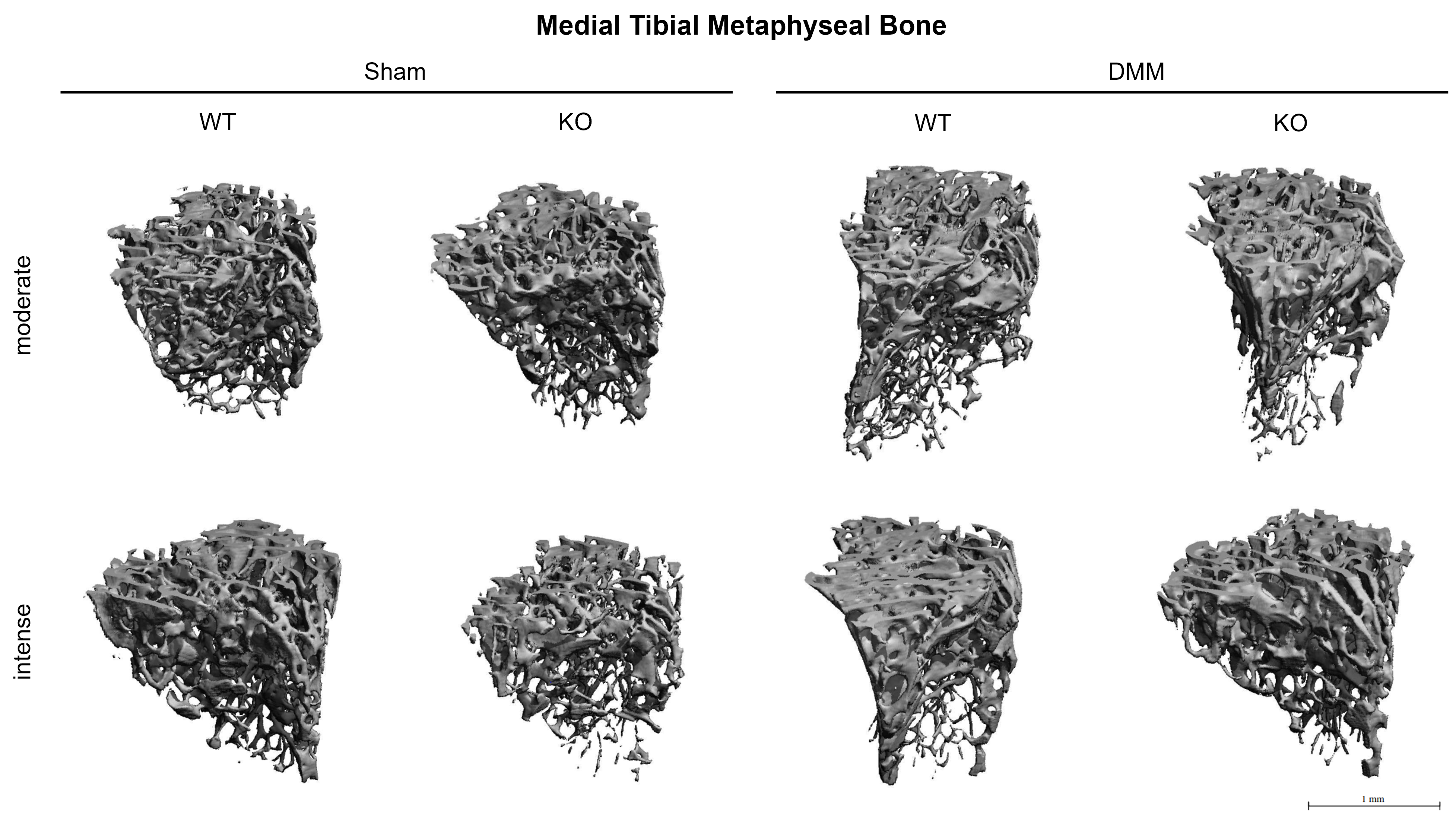

Supplement: Supplementary file 7 — Supplementary Material 7: Figure S7. Effect of Tac1 deficiency and forced exercise on metaphyseal bone morphology after OA-induction. Representative images of ultra-high resolution nanoCT analysis of the metaphyseal bone of the medial tibia in WT and KO mice exposed to moderate or intense exercise at 8 weeks after DMM or Sham surgery. [file 13075_2025_3693_MOESM7_ESM.png]
